# Supplementary material for: Prediction of 57Fe Mössbauer Nuclear Quadrupole Splittings with Hybrid and Double-Hybrid Density Functionals
Source: Int J Mol Sci. 2025 Mar 20;26(6):2821. doi: 10.3390/ijms26062821 (PMC11942716; doi:10.3390/ijms26062821)
Supplement: Supplementary file 1 [file ijms-26-02821-s001.zip › ijms-3514388-supplementary.pdf]

Supplementary Materials

Prediction of ^{57}Fe Mössbauer Nuclear Quadrupole Splittings with Hybrid and Double-Hybrid Density Functionals

Yihao Zhang, Haonan Tang, and Wenli Zou

March 12, 2025

Table S1: Theoretical V_c (in a.u.) of the Cu nucleus in CuF (r_e = 1.7449 Å). The experimental values is -0.425 a.u.

Ab initio	V_c	Error	Hybrid functional		V_c	Error	Double-hybrid functional		V_c	Error
HF	-1.252	-0.827		BH&HLYP	-0.391	0.034		ωB97X-2	0.023	0.448
CCSD	-0.553	-0.128		B3LYP	0.350	0.775		PWPB95	0.010	0.435
CCSD(T)	-0.449	-0.024		CAM-B3LYP	0.167	0.592	B2PLYP model	B2PLYP	0.104	0.529
CCSD(T) [a]	-0.433	-0.008		PW6B95	0.101	0.526		mPW2PLYP	0.002	0.427
				M06-2X	-0.313	0.112		B2GP-PLYP	-0.110	0.315
CASSCF(9o)	1.704	2.129		ωB97XD	0.184	0.609		B2K-PLYP	-0.231	0.194
MRCI(9o)	1.827	2.252	PBEn	PBE0	0.135	0.560		B2T-PLYP	-0.042	0.383
MRAQCC(9o)	1.724	2.149		PBE38	-0.191	0.234	DSD model	DSD-BLYP	-0.206	0.219
				PBE50	-0.489	-0.064		DSD-PBEP86	-0.176	0.249
CASSCF(14o)	-0.962	-0.537	TPSSn	TPSSh	0.395	0.820		DSD-PBEB95	-0.171	0.254
MRCI(14o) [b]	-0.603	-0.178		TPSS0	0.016	0.441	DH/QIDH model	PBE-0DH	-0.213	0.212
MRAQCC(14o) [b]	-0.367	0.058		TPSS38	-0.288	0.137		PBE-QIDH	-0.358	0.067
				TPSS50	-0.568	-0.143		RSX-0DH	-0.463	-0.038
			SCANn	SCAN0	-0.034	0.391		RSX-QIDH	-0.485	-0.060
				SCAN38	-0.383	0.042	LR corrected model	ωB2PLYP	-0.167	0.258
				SCAN50	-0.623	-0.198		ωB2GP-PLYP	-0.274	0.151
			rSCANn	rSCAN0	-0.094	0.331		ωB88PP86	-0.163	0.262
				rSCAN38	-0.351	0.074		ωPBEPP86	-0.229	0.196
				rSCAN50	-0.589	-0.164	DSD/DOD-2019 model	DOD-SCAN-D3(BJ)	-0.180	0.245
			r^2SCANn	r^2SCAN0	-0.116	0.309		noDispSD-SCAN69	-0.149	0.276
				r^2SCAN38	-0.370	0.055		revDSD-PBEP86-D3(BJ)	-0.126	0.299
				r^2SCAN50	-0.602	-0.177		revDSD-BLYP-D3(BJ)	-0.160	0.265
								revDOD-PBEP86-D3(BJ)	-0.137	0.288
							r^2SCAN-2023 model	r^2SCAN-0DH	-0.363	0.062
								r^2SCAN-CIDH	-0.398	0.027
								r^2SCAN-QIDH	-0.469	-0.044
								r^2SCAN0-2	-0.488	-0.063
								Pr^2SCAN50	-0.116	0.309
								Pr^2SCAN69	-0.313	0.112

[a] All the core electrons are correlated.

[b] Configurations with *weight* $\geq$ 0.01% are selected.

Table S2: Natural configuration of the Cu atom in CuF.

Method	Natural electron configuration
HF	$3d^{9.92}4s^{0.14}4p^{0.03}$
CCSD	$3d^{9.73}4s^{0.28}4p^{0.06}4d^{0.08}$
CCSD(T)	$3d^{9.70}4s^{0.30}4p^{0.07}4d^{0.09}$
CASSCF(9*o*)	$3d^{9.20}4s^{0.90}4p^{0.03}4d^{0.01}$
MRCI(9*o*)	$3d^{9.19}4s^{0.87}4p^{0.05}4d^{0.05}$
MRAQCC(9*o*)	$3d^{9.17}4s^{0.86}4p^{0.08}4d^{0.06}$
CASSCF(14*o*)	$3d^{9.80}4s^{0.23}4p^{0.03}4d^{0.07}$
MRCI(14*o*)	$3d^{9.75}4s^{0.25}4p^{0.05}4d^{0.08}$
MRAQCC(14*o*)	$3d^{9.71}4s^{0.28}4p^{0.06}4d^{0.08}$
BH&HLYP	$3d^{9.87}4s^{0.26}4p^{0.03}$
B3LYP	$3d^{9.81}4s^{0.39}4p^{0.04}$
B2PLYP (SCF part)	$3d^{9.87}4s^{0.26}4p^{0.03}$
B2PLYP	$3d^{9.72}4s^{0.38}4p^{0.05}4d^{0.06}$
r2SCAN-CIDH (SCF part)	$3d^{9.88}4s^{0.23}4p^{0.03}$
r2SCAN-CIDH	$3d^{9.81}4s^{0.30}4p^{0.04}4d^{0.03}$

Table S3: ΔE_Q results (in mm/s) of ^{57}Fe by selected hybrid functionals. [a)]

Mol. [b)]	$N_{\rm atom}^{2S+1}$	η [c)]	**H-1**	**H-2**	**H-3**	**H-4**	**H-5**	**H-6**	**H-7**	**H-8**	**H-9**	**H-10**	**H-11**	Expt.
**1***	42^2	0.50	-0.33	0.37	-0.32	-0.24	-0.35	-0.25	-0.50	-1.16	-0.30	-1.17	-0.30	-0.40
**2***	38^1	0.79	-0.47	-0.50	-0.43	-0.38	-0.47	-0.39	-0.47	-0.38	-0.46	-0.39	-0.47	0.29
**3***	44^1	0.79	-0.73	0.74	0.74	0.73	-0.75	0.72	-0.72	0.72	-0.73	0.72	-0.74	0.69
**4**	18^4	0.45	3.17	2.90	3.00	2.80	3.11	2.75	3.02	2.60	2.93	2.59	2.91	2.10
**5**	50^1	0.45	-0.90	-0.95	-0.94	-0.91	-0.90	-0.91	-0.90	-0.92	-0.92	-0.92	-0.91	-0.74
		0.44	-0.90	-0.94	-0.94	-0.91	-0.90	-0.89	-0.89	-0.91	-0.91	-0.91	-0.91	
**6**	59^1	0.34	-1.14	-1.12	-1.19	-1.19	-1.14	-1.17	-1.12	-1.20	-1.15	-1.20	-1.15	-1.14
**7**	17^5	0.06	-4.11	-4.01	-4.08	-4.05	-4.09	3.70	3.78	-4.05	-4.09	3.87	-4.09	-3.97
**8**	33^6	0.02	-0.34	-0.35	-0.33	-0.35	-0.32	-0.35	-0.33	-0.35	-0.33	-0.35	-0.32	-0.62
**9**	29^2	0.26	-0.77	-0.90	-0.78	-0.71	-0.80	-0.76	-0.83	-0.78	-0.84	-0.78	-0.84	-0.69
**10***	49^5	0.81	-3.65	-3.58	-3.57	-3.51	-3.59	3.31	3.40	-3.48	-3.56	-3.48	-3.55	-3.24
**11**	43^5	0.11	3.85	3.85	3.79	3.73	3.79	3.58	3.65	3.73	3.79	3.72	3.78	3.62
**12**	43^5	0.12	3.92	3.85	3.85	3.79	3.86	3.64	3.72	3.80	3.85	3.78	3.84	3.61
**13**	17^1	0.12	-1.59	-1.55	-1.59	-1.54	-1.57	-1.53	-1.57	-1.56	-1.59	-1.56	-1.59	-1.34
**14**	45^2	0.18	-2.04	-2.29	-2.01	-1.92	-1.86	-1.66	-1.65	-1.87	-1.82	-1.85	-1.79	-2.24
**15**	39^6	0.63	2.59	2.34	2.52	2.25	2.60	2.16	2.51	2.14	2.54	2.13	2.53	0.62
**16***	42^1	0.76	-1.56	-1.54	-1.59	-1.58	-1.51	-1.52	-1.48	-1.58	-1.53	-1.58	-1.52	-1.63
**17**	49^1	0.23	0.33	0.37	0.35	0.35	0.34	0.33	0.33	0.34	0.34	0.34	0.34	0.43
**18***	54^1	0.90	-0.59	-0.64	-0.59	-0.53	-0.59	-0.55	-0.59	-0.54	-0.59	-0.54	-0.59	0.48
**19**	71^1	0.64	0.49	0.45	0.51	0.52	0.47	0.53	0.48	-0.55	0.49	-0.54	0.49	-0.83
**20***	54^1	0.62	0.57	0.65	0.55	0.49	0.57	0.53	0.59	0.49	0.57	0.50	0.57	-0.35
**21**	44^1	0.38	0.97	1.03	0.97	0.92	0.96	0.94	0.97	0.94	0.97	0.94	0.97	0.73
**22**	27^1	0.58	1.13	1.20	1.13	1.09	1.12	1.09	1.11	1.10	1.13	1.10	1.13	0.89
**23***	44^5	0.98	-1.86	-1.85	-1.87	-1.82	-1.87	1.83	1.87	-1.84	-1.87	-1.84	-1.87	-1.42
**24**	58^3	0.48	-1.41	-1.27	-1.40	-1.10	-1.38	-1.21	-1.39	-1.19	-1.43	-1.20	-1.43	-1.24
**25**	25^4	0.49	-2.67	-2.68	-2.65	-2.66	-2.62	-2.58	-2.56	-2.67	-2.64	-2.68	-2.64	-2.05
**26**	21^1	0.00	4.14	4.14	4.01	3.62	4.01	3.80	4.09	3.65	4.03	3.65	4.03	2.41
**27**	13^6	0.42	-0.87	-0.82	-0.85	-0.90	-0.85	-0.87	-0.83	-0.87	-0.83	-0.87	-0.83	-1.23
**28**	22^2	0.49	-1.29	-1.42	-1.29	-1.22	-1.30	-1.28	-1.35	-1.29	-1.35	-1.29	-1.35	-1.12
**29**	51^2	0.11	0.77	0.59	0.79	0.68	0.74	0.70	0.73	0.81	0.89	0.81	0.89	0.89
**30**	21^5	0.13	1.96	1.96	1.89	1.84	1.90	1.67	1.76	1.79	1.87	1.77	1.86	1.80
**31**	56^5	0.34	2.63	2.64	2.56	2.50	2.57	2.34	2.44	2.46	2.55	2.45	2.54	2.10
**32**	25^5	0.73	2.75	2.74	2.68	2.63	2.69	2.48	2.56	2.60	2.67	2.59	2.66	2.36
MaxE [d)]			1.73	1.73	1.60	1.35	1.60	7.67	7.75	1.24	1.62	7.84	1.62	
MAE [d)]			0.33	0.33	0.31	0.27	0.32	0.50	0.54	0.25	0.32	0.49	0.32	
MAE [e)]			0.30	0.30	0.28	0.24	0.29	0.24	0.28	0.25	0.28	0.25	0.28	

[a)] The hybrid functionals are BH&HLYP (**H-1**), M06-2X (**H-2**), PBE50 (**H-3**), TPSS38 (**H-4**), TPSS50 (**H-5**), SCAN38 (**H-6**), SCAN50 (**H-7**), *r*SCAN38 (**H-8**), *r*SCAN50 (**H-9**), r^2SCAN38 (**H-10**), and r^2SCAN50 (**H-11**).

[b)] The molecule with an asterisk means that the sign of ΔE_Q is theoretically uncertain and therefore $|\Delta E_Q|$ is used for error analysis.

[c] Calculated by r^2SCAN-CIDH.
[d] Maximum error and mean absolute error. Molecule **15** has been excluded.
[e] Mean absolute error of $|\Delta E_Q|$. Molecule **15** has been excluded.

Table S4: ΔE_Q results (in mm/s) of ^{57}Fe by selected double-hybrid functionals. [a)]

Mol. [b)]	N^{2S+1}_{atom}	Density [c)]	η [d)]	**DH-1**	**DH-2**	**DH-3**	**DH-4**	**DH-5**	**DH-6**	**DH-7**	**DH-8**	**DH-9**	**DH-10**	**DH-11**	**DH-12**	Expt.
**1***	42^2	HFun		-0.33	0.34	-0.32	-0.53	-0.53	-0.52	-0.50	-0.49	-0.47	-0.48	-0.32	-0.43	
		UnRlx		-0.31	0.33	-0.30	-0.49	-0.49	-0.49	-0.47	-0.45	-0.43	-0.44	-0.29	-0.42	
		Rlx	0.50	-0.44	-1.01	0.33	0.23	-0.74	0.23	-0.72	-0.89	-0.80	-1.13	-0.32	0.24	-0.40
**2***	38^1	HFun		-0.52	-0.68	-0.48	-0.65	-0.67	-0.65	-0.64	-0.62	-0.60	-0.64	-0.44	-0.56	
		UnRlx		-0.48	-0.63	-0.45	-0.59	-0.60	-0.60	-0.60	-0.56	-0.54	-0.57	-0.41	-0.54	
		Rlx	0.79	-0.29	-0.33	-0.29	0.20	0.19	-0.27	-0.31	0.18	0.21	0.18	0.21	-0.40	0.29
**3***	44^1	HFun		-0.73	-0.73	-0.73	-0.73	-0.73	-0.75	-0.76	-0.75	-0.74	-0.73	0.74	-0.78	
		UnRlx		-0.73	-0.73	-0.73	-0.73	-0.73	-0.75	-0.75	-0.75	-0.73	-0.73	0.73	-0.78	
		Rlx	0.79	0.74	0.77	0.73	0.78	0.79	0.79	0.80	0.82	0.78	0.79	0.74	0.79	0.69
**4**	18^4	HFun		2.97	3.45	2.87	3.48	3.51	3.49	3.45	3.37	3.34	3.35	2.99	3.33	
		UnRlx		2.99	3.49	2.88	3.51	3.55	3.52	3.47	3.41	3.36	3.38	2.99	3.34	
		Rlx	0.45	3.28	3.70	3.10	3.16	3.10	3.24	3.25	3.07	3.07	3.20	2.69	3.20	2.10
**5**	50^1	HFun		-0.89	0.81	-0.90	0.81	0.80	0.84	0.85	0.85	0.86	0.83	-0.94	-0.92	
				-0.88	0.80	-0.89	0.81	0.80	0.83	0.85	0.85	0.85	0.82	-0.93	-0.91	
		UnRlx		-0.91	0.83	-0.91	-0.85	-0.84	-0.86	-0.88	-0.89	-0.89	-0.87	-0.95	-0.93	
				-0.90	0.83	-0.91	-0.84	-0.83	-0.86	-0.87	-0.88	-0.88	-0.86	-0.94	-0.92	
		Rlx	0.45	-0.98	-1.07	-0.95	-1.17	-1.23	-1.09	-1.07	-1.23	-1.13	-1.21	-0.98	-0.96	-0.74
			0.44	-0.97	-1.07	-0.95	-1.16	-1.22	-1.08	-1.06	-1.22	-1.12	-1.20	-0.98	-0.96	
**6**	59^1	HFun		-1.11	-0.94	-1.14	-0.98	-0.96	-1.02	-1.03	-1.03	-1.04	-1.00	-1.18	-1.15	
		UnRlx		-1.13	-0.99	-1.16	-1.03	-1.02	-1.06	-1.07	-1.09	-1.08	-1.06	-1.20	-1.17	
		Rlx	0.34	-1.36	-1.47	-1.32	-1.67	-1.76	-1.56	-1.51	-1.78	-1.61	-1.74	-1.39	-1.32	-1.14
**7**	17^5	HFun		-4.10	-4.10	-4.09	-4.11	-4.11	-4.12	-4.11	-4.10	-4.10	-4.09	-4.08	-4.11	
		UnRlx		-4.12	-4.16	-4.10	-4.17	-4.19	-4.18	-4.15	-4.17	-4.15	-4.16	-4.10	-4.13	
		Rlx	0.06	-4.06	-4.10	-4.05	-4.12	-4.14	-4.13	-4.12	-4.12	-4.10	-4.10	-4.04	-4.11	-3.97
**8**	33^6	HFun		-0.31	-0.26	-0.32	-0.28	-0.27	-0.29	-0.28	-0.28	-0.28	-0.27	-0.33	-0.32	
		UnRlx		-0.31	-0.26	-0.32	-0.29	-0.28	-0.30	-0.28	-0.29	-0.29	-0.28	-0.34	-0.32	
		Rlx	0.02	-0.35	-0.34	-0.36	-0.39	-0.39	-0.38	-0.36	-0.40	-0.37	-0.39	-0.39	-0.35	-0.62
**9**	29^2	HFun		-0.89	-1.00	-0.86	-0.94	-0.96	-0.91	-0.93	-0.92	-0.92	-0.95	-0.79	-0.83	
		UnRlx		-0.88	-0.99	-0.85	-0.93	-0.95	-0.90	-0.93	-0.91	-0.92	-0.94	-0.79	-0.83	
		Rlx	0.26	-0.76	-0.82	-0.75	-0.71	-0.70	-0.71	-0.77	-0.67	-0.73	-0.70	-0.67	-0.75	-0.69
**10***	49^5	HFun		-3.59	-3.67	-3.56	-3.72	-3.72	-3.72	-3.67	-3.66	-3.66	-3.67	-3.57	-3.65	
		UnRlx		-3.60	-3.72	-3.57	-3.77	-3.78	-3.76	-3.71	-3.72	-3.70	-3.73	-3.59	-3.66	
		Rlx	0.81	-3.50	-3.59	-3.48	-3.59	-3.59	-3.61	-3.59	-3.53	-3.55	-3.54	-3.47	-3.60	-3.24
**11**	43^5	HFun		3.80	3.85	3.78	3.89	3.89	3.90	3.85	3.85	3.84	3.86	3.79	3.84	
		UnRlx		3.81	3.91	3.79	3.94	3.96	3.95	3.90	3.91	3.88	3.91	3.81	3.86	
		Rlx	0.11	3.73	3.79	3.72	3.83	3.84	3.85	3.81	3.78	3.78	3.78	3.71	3.81	3.62
**12**	43^5	HFun		3.86	3.91	3.84	3.94	3.94	3.97	3.92	3.92	3.90	3.92	3.86	3.91	
		UnRlx		3.88	3.96	3.86	3.99	4.00	4.01	3.96	3.98	3.95	3.97	3.87	3.93	
		Rlx	0.12	3.79	3.85	3.78	3.89	3.91	3.92	3.89	3.86	3.84	3.85	3.77	3.88	3.61
**13**	17^1	HFun		-1.59	-1.53	-1.58	-1.55	-1.54	-1.61	-1.61	-1.59	-1.57	-1.55	-1.59	-1.69	

(*Continued on next page.*)

Table S4: *Continued from previous page.*

Mol. [b]	N^{2S+1}_{atom}	Density [c]	η [d]	**DH-1**	**DH-2**	**DH-3**	**DH-4**	**DH-5**	**DH-6**	**DH-7**	**DH-8**	**DH-9**	**DH-10**	**DH-11**	**DH-12**	Expt.
		UnRlx		-1.57	-1.51	-1.56	-1.52	-1.51	-1.60	-1.60	-1.57	-1.55	-1.52	-1.56	-1.68	
		Rlx	0.12	-1.68	-2.29	-1.63	-2.43	-2.70	-2.18	-2.09	-2.40	-2.12	-2.46	-1.64	-1.75	-1.34
**14**	45^2	HFun		-1.73	-1.69	-1.78	-1.84	-1.81	-1.94	-1.88	-1.86	-1.84	-1.82	-2.00	-2.06	
		UnRlx		-1.75	-1.72	-1.79	-1.88	-1.85	-1.97	-1.90	-1.90	-1.87	-1.85	-2.01	-2.07	
		Rlx	0.18	-2.03	-4.21	-1.99	-2.43	-2.53	-2.43	-2.34	-2.62	-2.37	-2.52	-2.19	-2.24	-2.24
**15**	39^6	HFun		2.63	2.81	2.52	2.94	2.85	2.93	2.80	2.89	2.86	2.89	2.51	2.79	
		UnRlx		2.64	2.86	2.52	2.98	2.90	2.97	2.83	2.94	2.89	2.94	2.52	2.80	
		Rlx	0.63	2.33	2.75	2.25	2.43	2.67	2.52	2.66	2.34	2.39	2.33	2.06	2.59	0.62
**16***	42^1	HFun		-1.45	-1.19	-1.50	-1.27	-1.24	-1.33	-1.30	-1.31	-1.36	-1.29	-1.58	-1.50	
		UnRlx		-1.51	-1.28	-1.54	-1.38	-1.37	-1.41	-1.38	-1.42	-1.45	-1.41	-1.63	-1.53	
		Rlx	0.76	2.05	2.62	1.93	2.99	3.25	2.58	2.38	3.05	2.71	3.13	2.06	1.85	-1.63
**17**	49^1	HFun		0.33	0.31	0.33	0.31	0.31	0.32	0.33	0.33	0.32	0.32	0.34	0.35	
		UnRlx		0.33	0.32	0.34	0.32	0.32	0.33	0.33	0.34	0.33	0.32	0.35	0.35	
		Rlx	0.23	0.36	0.37	0.36	0.39	0.41	0.39	0.39	0.43	0.40	0.41	0.38	0.38	0.43
**18***	54^1	HFun		-0.63	-0.77	-0.60	-0.74	-0.76	-0.74	-0.74	-0.73	-0.71	-0.74	-0.59	-0.68	
		UnRlx		-0.60	-0.72	-0.57	-0.67	-0.68	-0.69	-0.70	-0.66	-0.65	-0.67	-0.56	-0.66	
		Rlx	0.90	-0.50	-0.52	-0.50	0.56	0.59	0.54	-0.53	0.61	0.54	0.58	0.50	-0.56	0.48
**19**	71^1	HFun		0.45	0.38	0.47	0.39	0.39	0.41	0.41	0.42	0.42	0.40	0.50	0.47	
		UnRlx		0.47	0.37	0.48	0.39	0.38	0.41	0.41	0.43	0.42	0.40	0.52	0.48	
		Rlx	0.64	-0.69	-0.70	-0.67	-0.92	-0.99	-0.83	-0.75	-1.03	-0.87	-0.97	-0.76	-0.62	-0.83
**20***	54^1	HFun		0.62	0.80	0.58	0.76	0.78	0.75	0.75	0.74	0.71	0.75	0.56	0.67	
		UnRlx		0.58	0.74	0.55	0.69	0.70	0.70	0.71	0.67	0.65	0.67	0.52	0.65	
		Rlx	0.62	0.38	0.41	0.39	0.34	0.35	0.37	0.41	0.36	0.34	0.34	0.36	0.50	-0.35
**21**	44^1	HFun		0.98	0.99	0.98	1.00	1.00	1.01	1.01	1.00	0.99	1.00	0.97	1.01	
		UnRlx		0.98	0.99	0.97	0.99	0.99	1.01	1.00	1.00	0.99	0.99	0.96	1.01	
		Rlx	0.38	0.92	1.02	0.90	0.99	1.01	0.98	0.99	0.99	0.96	0.99	0.86	0.97	0.73
**22**	27^1	HFun		1.13	1.12	1.13	1.12	1.12	1.15	1.15	1.14	1.14	1.13	1.13	1.18	
		UnRlx		1.13	1.12	1.13	1.12	1.12	1.15	1.15	1.14	1.13	1.13	1.13	1.18	
		Rlx	0.58	1.10	1.21	1.08	1.18	1.21	1.18	1.19	1.19	1.15	1.19	1.04	1.15	0.89
**23***	44^5	HFun		-1.87	-1.93	-1.87	-1.92	-1.92	-1.94	-1.95	-1.92	-1.91	-1.92	-1.87	-1.95	
		UnRlx		-1.86	-1.92	-1.87	-1.91	-1.91	-1.94	-1.94	-1.91	-1.90	-1.91	-1.86	-1.95	
		Rlx	0.98	-1.83	-1.89	-1.85	-1.83	-1.81	-1.87	-1.89	-1.83	-1.84	-1.83	-1.82	-1.93	-1.42
**24**	58^3	HFun		-1.51	1.64	-1.44	1.57	1.60	1.58	1.63	1.61	1.59	1.60	-1.40	-1.54	
		UnRlx		-1.51	1.63	-1.43	1.57	1.60	-1.58	1.63	-1.60	1.58	1.59	-1.38	-1.54	
		Rlx	0.48	-1.37	-1.67	-1.26	-1.46	-1.56	-1.43	-1.53	-1.59	-1.47	-1.54	-1.15	-1.35	-1.24
**25**	25^4	HFun		-2.61	1.50	-2.63	-2.58	-2.57	-2.64	-2.60	-2.59	-2.57	1.54	-2.64	-2.71	
		UnRlx		-2.62	1.48	-2.64	-2.60	-2.59	-2.65	-2.62	-2.62	-2.59	1.53	-2.65	-2.72	
		Rlx	0.49	-2.73	1.79	-2.72	-2.96	-3.15	-2.86	-2.81	-3.03	-2.84	1.84	-2.76	-2.80	-2.05
**26**	21^1	HFun		4.20	4.84	4.05	4.75	4.82	4.69	4.69	4.66	4.58	4.69	4.02	4.34	
		UnRlx		4.11	4.74	3.97	4.60	4.65	4.59	4.60	4.51	4.44	4.53	3.91	4.29	

(*Continued on next page.*)

Table S4: *Continued from previous page.*

Mol. [b]	N_{atom}^{2S+1}	Density [c]	η [d]	**DH-1**	**DH-2**	**DH-3**	**DH-4**	**DH-5**	**DH-6**	**DH-7**	**DH-8**	**DH-9**	**DH-10**	**DH-11**	**DH-12**	Expt.
		Rlx	0.00	3.08	2.93	3.12	2.26	1.97	2.68	2.94	1.97	2.46	2.01	2.78	3.52	2.41
**27**	13^6	HFun		-0.82	-0.77	-0.83	-0.79	-0.79	-0.80	-0.79	-0.79	-0.79	-0.78	-0.85	-0.83	
		UnRlx		-0.82	-0.78	-0.83	-0.80	-0.80	-0.81	-0.80	-0.80	-0.80	-0.79	-0.85	-0.84	
		Rlx	0.42	-0.86	-0.84	-0.87	-0.90	-0.90	-0.90	-0.86	-0.91	-0.88	-0.89	-0.92	-0.87	-1.23
**28**	22^2	HFun		-1.39	-1.50	-1.36	-1.44	-1.46	-1.42	-1.43	-1.42	-1.42	-1.45	-1.30	-1.35	
		UnRlx		-1.39	-1.49	-1.36	-1.45	-1.46	-1.42	-1.44	-1.43	-1.43	-1.45	-1.31	-1.35	
		Rlx	0.49	-1.27	-1.32	-1.25	-1.25	-1.25	-1.25	-1.29	-1.22	-1.26	-1.24	-1.19	-1.27	-1.12
**29**	51^2	HFun		0.91	0.95	0.87	0.82	0.81	0.77	0.77	0.84	0.88	0.91	0.78	0.77	
		UnRlx		0.95	1.01	0.91	0.89	0.88	0.83	0.83	0.91	0.93	0.98	0.81	0.79	
		Rlx	0.11	0.43	-0.48	0.61	0.45	0.63	0.69	0.69	0.39	0.26	-0.13	0.53	0.66	0.89
**30**	21^5	HFun		1.88	1.94	1.86	1.99	1.99	1.99	1.95	1.96	1.94	1.96	1.89	1.93	
		UnRlx		1.90	2.00	1.87	2.05	2.06	2.04	1.99	2.02	1.99	2.02	1.90	1.95	
		Rlx	0.13	1.79	1.85	1.77	1.89	1.90	1.91	1.89	1.86	1.85	1.85	1.78	1.88	1.80
**31**	56^5	HFun		2.66	2.58	2.55	2.70	2.70	2.70	2.65	2.66	2.64	2.67	2.56	2.62	
		UnRlx		2.71	2.59	2.56	2.75	2.76	2.74	2.69	2.72	2.68	2.72	2.58	2.64	
		Rlx	0.34	2.54	2.47	2.45	2.56	2.56	2.58	2.56	2.51	2.51	2.51	2.44	2.56	2.10
**32**	25^5	HFun		2.69	2.76	2.67	2.79	2.80	2.79	2.75	2.76	2.74	2.77	2.69	2.73	
		UnRlx		2.70	2.80	2.67	2.84	2.85	2.83	2.79	2.81	2.78	2.82	2.70	2.74	
		Rlx	0.73	2.60	2.65	2.58	2.67	2.67	2.69	2.67	2.62	2.63	2.63	2.58	2.67	2.36
MaxE [e]		HFun		1.79	3.55	1.64	2.81	2.84	2.82	2.87	2.85	2.83	3.59	1.61	1.93	
		UnRlx		1.70	3.53	1.56	2.81	2.84	2.18	2.87	2.10	2.82	3.58	1.50	1.88	
		Rlx		1.18	3.84	1.00	1.36	1.62	1.14	1.15	1.42	1.08	3.89	-0.71	1.11	
MAE [e]		HFun		0.34	0.69	0.32	0.58	0.59	0.59	0.58	0.57	0.56	0.67	0.31	0.37	
		UnRlx		0.34	0.68	0.31	0.48	0.49	0.41	0.48	0.40	0.46	0.57	0.31	0.37	
		Rlx		0.25	0.54	0.23	0.34	0.39	0.31	0.31	0.37	0.31	0.49	0.20	0.28	
MAE [f]		Rlx		0.25	0.40	0.23	0.34	0.39	0.31	0.31	0.37	0.31	0.36	0.20	0.28	

[a] The double-hybrid functionals are r^2SCAN-CIDH (**DH-1**), r^2SCAN0-2 (**DH-2**), r^2SCAN-0DH (**DH-3**), B2K-PLYP (**DH-4**), DSD-BLYP (**DH-5**), ωB2GP-PLYP (**DH-6**), RSX-QIDH (**DH-7**), ωPBEPP86 (**DH-8**), PBE-QIDH (**DH-9**), DSD-PBEP86 (**DH-10**), PBE-0DH (**DH-11**), and RSX-0DH (**DH-12**).

[b] The molecule with an asterisk means that the sign of ΔE_Q is theoretically uncertain and therefore $|\Delta E_Q|$ is used for error analysis.

[c] HFun: density of truncated hybrid functional. UnRlx: unrelaxed PT2 density. Rlx: relaxed PT2 density.

[d] Calculated by r^2SCAN-CIDH.

[e] Maximum error and mean absolute error. Molecule **15** has been excluded.

[f] Mean absolute error of $|\Delta E_Q|$. Molecule **15** has been excluded.

Table S5: ΔE_Q results (in mm/s) of ^{57}Fe by standard and augmented basis functions. [a)]

Func. [b)]	Mol.	Expt.	S-BAS	A-BAS	Diff.	Mol.	Expt.	S-BAS	A-BAS	Diff.
	**13**	-1.34				**30**	1.80			
**H-1**			-1.594	-1.586	0.008			1.957	1.943	-0.014
**H-2**			-1.549	-1.530	0.019			1.959	1.954	-0.005
**H-3**			-1.592	-1.586	0.006			1.886	1.873	-0.013
**H-4**			-1.545	-1.538	0.007			1.837	1.822	-0.015
**H-5**			-1.568	-1.563	0.005			1.896	1.879	-0.017
**H-6**			-1.530	-1.522	0.008			1.675	1.657	-0.018
**H-7**			-1.566	-1.558	0.008			1.756	1.740	-0.016
**H-8**			-1.563	-1.555	0.008			1.787	1.772	-0.015
**H-9**			-1.591	-1.586	0.005			1.869	1.858	-0.011
**H-10**			-1.563	-1.556	0.007			1.775	1.763	-0.012
**H-11**			-1.592	-1.584	0.008			1.861	1.847	-0.014
**DH-1**			-1.684	-1.677	0.007			1.789	1.776	-0.013
**DH-2**			-2.286	-2.282	0.004			1.853	1.840	-0.013
**DH-3**			-1.627	-1.619	0.008			1.772	1.758	-0.014
**DH-4**			-2.429	-2.427	0.002			1.892	1.880	-0.012
**DH-5**			-2.704	-2.701	0.003			1.901	1.890	-0.011
**DH-6**			-2.181	-2.179	0.002			1.909	1.897	-0.012
**DH-7**			-2.095	-2.091	0.004			1.887	1.874	-0.013
**DH-8**			-2.404	-2.400	0.004			1.856	1.844	-0.012
**DH-9**			-2.123	-2.119	0.004			1.849	1.837	-0.012
**DH-10**			-2.459	-2.457	0.002			1.850	1.839	-0.011
**DH-11**			-1.636	-1.630	0.006			1.781	1.769	-0.012
**DH-12**			-1.750	-1.742	0.008			1.885	1.871	-0.014

[a)] The standard basis functions (S-BAS) may be found in the Computational Methods subsection in the main text, whereas in the augmented basis functions (A-BAS) the iron atom are added with two tighter and two more diffuse Gaussian functions with $\alpha_p = 26521.473$, 0.0311738 and $\alpha_d = 6191.1311$, 0.0295094.

[b)] The hybrid and double-hybrid functionals have been defined in Tables S3 and S4.
